# O-GlcNAcylation expression predicts a favorable prognosis and mitigates malignant phenotypes via MYCN suppression in neuroblastoma

**DOI:** 10.1186/s40348-026-00218-3

**Published:** 2026-02-10

**Authors:** Neng-Yu Lin, Hsiu-Hao Chang, Chia-Yeh Hsieh, Hsiu-Ling Chang, Wan-Ling Ho, Yen-Lin Liu, Pei-Yi Wu, Chi-Tai Yeh, Min-Chuan Huang, Wen-Ming Hsu

**Affiliations:** 1 Graduate Institute of Anatomy and Cell Biology, National Taiwan University College of Medicine, Taipei, Taiwan; 2 Department of Pediatrics, National Taiwan University Hospital, National Taiwan University College of Medicine, Taipei, Taiwan; 3 Department of Pediatrics, Taipei Medical University Hospital, Taipei, Taiwan; 4 Department of Pediatrics, School of Medicine, College of Medicine, Taipei Medical University, Taipei, Taiwan; 5 Department of Life Sciences, National Central University, Taoyuan, Taiwan; 6 Department of Medical Research and Education, Taipei Medical University Shuang-Ho Hospital, New Taipei City, 23561 Taiwan; 7 Continuing Education Program of Food Biotechnology Applications, College of Science and Engineering, National Taitung University, Taitung, 95092 Taiwan; 8 Department of Surgery, National Taiwan University Hospital, National Taiwan University College of Medicine, No. 8 Chung-Shan South Road, Taipei, 10041 Taiwan

**Keywords:** O-GlcNAc, Neuroblastoma, MYCN, GSK3β

## Abstract

**Background:**

Neuroblastoma (NB) is a common pediatric malignancy originating from neural crest progenitor cells. While O-GlcNAcylation is known to regulate cancer cell metabolism and behavior, its specific role and prognostic value in neuroblastoma remain poorly understood. This study aims to elucidate the clinical significance and molecular mechanisms of O-GlcNAcylation in NB.

**Methods:**

We analyzed O-GlcNAcylated protein expression in 158 human NB tumor samples using immunohistochemistry (IHC) and correlated the findings with clinicopathological parameters and survival outcomes. The therapeutic potential of enhancing O-GlcNAcylation via the OGA inhibitor Thiamet G was evaluated in MYCN-amplified NB cell lines and the *Th-MYCN* transgenic mouse model. Molecular mechanisms governing MYCN stability were investigated using Western blotting, immunoprecipitation, and functional assays.

**Results:**

High levels of O-GlcNAcylated proteins were significantly associated with differentiated histology and early clinical stages. Survival analysis identified high O-GlcNAc expression as an independent prognostic factor for favorable outcomes. In vitro and in vivo experiments demonstrated that Thiamet G treatment effectively suppressed tumor growth and invasion while promoting neuronal differentiation. Mechanistically, Thiamet G-induced O-GlcNAc accumulation reduced inhibitory phosphorylation of GSK3β at Ser9, thereby activating GSK3β. This activation promoted the phosphorylation of MYCN at Thr58, accelerating its degradation via the ubiquitin-proteasome pathway.

**Conclusion:**

Our findings demonstrate that high O-GlcNAcylated protein levels predict a favorable prognosis in neuroblastoma. Pharmacological inhibition of OGA with Thiamet G destabilizes the MYCN oncoprotein via the GSK3β-proteasome axis, suppressing tumorigenesis and inducing differentiation. This suggests that modulating O-GlcNAc levels represents a promising therapeutic strategy for MYCN-driven neuroblastoma.

**Supplementary Information:**

The online version contains supplementary material available at 10.1186/s40348-026-00218-3.

## Introduction

Neuroblastoma (NB) is a major pediatric cancer, being the most common extracranial solid tumor in children and infants. It originates from the sympathoadrenal lineage of neural crest progenitor cells, forming in neurons of the sympathetic nervous system and the adrenal gland’s medulla. Each year, about 700–800 cases are diagnosed in the United States [1] (6% of childhood cancers) and around 30 cases in Taiwan (4–6% of pediatric cancers). Although NB represents 5–8% of malignant childhood diseases, it disproportionately causes up to 10–15% of cancer deaths in children [2]. The clinical presentation of NB can be categorized into three cell types based on differentiation: neuroblastic (poorly differentiated) tumors, ganglioneuroblastomas (GNB, intermixed), and ganglioneuromas (GN, well-differentiated) [3]. Neuroblastic tumors consist mainly of undifferentiated neuroblasts and are aggressive with a poor prognosis, often linked to MYCN amplification. Ganglioneuroblastomas, with a mix of neuroblasts and ganglion cells, show intermediate prognosis. Ganglioneuromas, composed predominantly of mature ganglion cells and Schwannian stroma, have a favorable prognosis and are slow-growing [4]. These distinctions are crucial for treatment strategies and prognosis prediction in neuroblastoma.

Current multimodal therapeutic strategies for high-risk neuroblastoma include intensive induction chemotherapy, surgery, high-dose chemotherapy with autologous stem cell rescue, radiotherapy, and maintenance therapy with anti-GD2 immunotherapy and retinoic acid (RA) [5]. Despite these intensive treatments, the prognosis for high-risk patients remains poor, largely driven by oncogenic factors. MYCN proto-oncogene amplification, detected in 20–30% of cases, strongly indicates aggressive tumor behavior and resistance to treatment in neuroblastoma (NB) [4, 6–8]. Beyond amplification, MYCN expression in NB is tightly regulated through diverse mechanisms. Significantly, post-translational modifications such as phosphorylation, acetylation, ubiquitination, and glycosylation influence MYCN stability, localization, and interactions with regulatory proteins in NB [9–12]. Furthermore, the degradation pathways mediated by glycogen synthase kinase 3 beta (GSK3β) regulate MYCN levels, influencing tumor aggressiveness, and are suppressed by phosphatidylinositol 3-kinase (PI3K) and AKT kinase signaling [13, 14]. Insight into these mechanisms is essential for devising targeted therapies to modulate MYCN function and improve treatment efficacy in neuroblastoma patients.

Glycosylation, a prevalent post-translational modification of proteins in mammalian cells, is crucial in regulating cancer development and progression [15]. Among these modifications, O-GlcNAcylation involves the dynamic addition or removal of a single O-linked N-acetylglucosamine (O-GlcNAc) moiety from serine or threonine residues of nucleocytoplasmic proteins [16]. This process is catalyzed by two enzymes with opposing functions: O-GlcNAc transferase (OGT) transfers the GlcNAc moiety from UDP-GlcNAc to serine or threonine residues of target proteins, while O-GlcNAcase (OGA) catalyzes the hydrolysis of the sugar to remove it. Together, they maintain finely balanced cellular O-GlcNAcylation levels through buffering regulation. Mass spectrometry has identified thousands of O-GlcNAcylated proteins with diverse functions, many of which are also phosphoproteins. O-GlcNAcylation and phosphorylation can interact, influencing each other at the same or nearby sites [17]. This modification acts as a nutrient sensor, integrating cellular signaling with metabolic pathways like those involving glucose and acetyl-CoA regulation [18–20]. Dysregulated O-GlcNAcylation has been implicated in diseases such as diabetes, neurodegenerative diseases, and cancer [21–23]. However, its specific role in pediatric cancers like neuroblastoma remains unclear.

The current study is the first to demonstrate that elevated O-GlcNAc expression correlates with the differentiation status of NB tumors and predicts favorable survival outcomes in NB patients. Extensive in vitro and in vivo experiments demonstrated that pharmacological inhibition of OGA activity with Thiamet G, a selective small-molecule inhibitor widely used in preclinical research, enhanced NB cell differentiation, suppressed malignant behaviors, and facilitated MYCN degradation via GSK3β activation. Importantly, this treatment significantly reduced tumor growth and promoted neuronal differentiation in preclinical neuroblastoma models. These findings suggest promising avenues for therapeutic intervention in neuroblastoma by regulating O-GlcNAcylation pathways to improve patient outcomes.

## Materials and methods

### Public database analysis

To evaluate the prognostic value of *OGT* and *OGA* mRNA expression, we utilized the Kocak neuroblastoma dataset (Accession No. GSE45547), which is publicly available via the R2: Genomics Analysis and Visualization Platform (http://r2.amc.nl) [24]. This dataset comprises single-color gene expression profiles from 649 neuroblastoma tumors generated using 44 K oligonucleotide microarrays (Agilent Technologies). The data were accessed using the R2 internal identifier ‘ps_avgpres_gse45547geo649_ag44kcwolf’. Gene expression data were pre-processed and normalized through the R2 platform pipeline. Clinical characteristics, including International Neuroblastoma Staging System (INSS) stage, MYCN amplification status, and survival outcomes, were included in the analysis to determine associations with OGT and OGA expression patterns.

### Patients and treatment

This study included 158 patients with histologically confirmed neuroblastoma (NB) and complete follow-up data obtained from the National Taiwan University Hospital (NTUH), covering the period from December 1990 to December 2020. The median age of the patients at diagnosis was 2.9 years (range, 0.5 months to 32.9 years). Tumor diagnoses were verified through histologic assessment of specimens obtained from primary or metastatic sites during surgery. According to the International NB Pathology Classification [25], tumors were categorized based on differentiation status: undifferentiated NB (UNB, Schwannian stroma-poor), differentiating NB (DNB, Schwannian stroma-poor, including poorly differentiated subtype), and ganglioneuroblastoma, intermixed (GNB, Schwannian stroma-rich). The nodular type GNB was further classified as either undifferentiated or differentiating NB, based on the morphology of their nodules, as prognosis mainly depends on the nodules [26]. Tumor staging followed the International NB Staging System criteria [27]. The MYCN status of tumor tissue was assessed using chromogenic in situ hybridization, which analyzed either formalin-fixed paraffin-embedded tissues or freshly isolated individual tumor cells [28]. The treatment of these patients has been described in previous studies [29, 30]. Clinical parameters, including age at diagnosis, sex, primary tumor sites, clinical stages, tumor histology differentiation status, and MYCN amplification status, were collected for prognostic and survival analysis. This study was approved by the Institutional Review Board of the National Taiwan University Hospital (approval no. 202108106RINB).

### Immunohistochemistry

One hundred and fifty-eight tumor specimens from NB patients, collected pre-chemotherapy, and neuroblastoma tumors from MYCN transgenic mice were fixed in formalin (Sigma) and embedded in paraffin (Sigma) for immunohistochemical staining. O-GlcNAcylated protein expression was assessed using an avidin-biotin complex immunoperoxidase staining method, as previously described [31]. Monoclonal anti-O-GlcNAc antibody (Clone RL2, Cat. No. MA1-072, Invitrogen; Thermo Fisher Scientific, Inc., dilution at 1:200) was employed and detected using the Super Sensitive Link-Label immunohistochemistry Detection System (BioGenex). Specific staining was visualized with the 3,3-diaminobenzidine liquid substrate system (Sigma) and counterstained with hematoxylin (Sigma). Slides were evaluated and photographed using a Nexcope NE950 fluorescent microscope at 200× and 400× magnification. O-GlcNAc immunoreactivity was scored based on the percentage of positive neuroblastoma cells relative to the total number of neuroblastoma cells (excluding stromal components). The expression levels were categorized into four levels: “−” (no expression), “1+” (weak, 10–35% of cells stained), “2+” (moderate, 35–70% of cells stained), and “3+” (strong, > 70% of cells stained). For statistical analysis, tumors were grouped into low expression (“−” or “1+”) and high expression (“2+” or “3+”).

### Cell lines and cell culture

Human NB cells, GI-ME-N, IMR-32, NMB, SH-SY5Y, SK-N-AS, SK-N-BE, SK-N-SH and SK-N-DZ, were cultured with Dulbecco’s modified Eagle’s medium (DMEM; Invitrogen) containing 10% FBS (Invitrogen), 100 IU/mL penicillin, and 100 µg/mL streptomycin (Invitrogen) in a humidified tissue culture incubator at 37 °C and 5% CO2 atmosphere. Regular mycoplasma contamination screening was conducted every three months using the Mycoplasma Detection Kit (D101, Vazyme Biotech Co., Ltd). Drug Treatments For in vitro experiments, cells were treated with the following compounds: Retinoic acid (RA; Sigma-Aldrich, Cat. No. R2625), Thiamet-G (Selleckchem, Cat. No. S7213), Cycloheximide (CHX; Sigma-Aldrich, Cat. No. C4859), and MG132 (Sigma-Aldrich, Cat. No. 474790). Unless otherwise specified, Thiamet-G was used at concentrations ranging from 5 to 20 µM; Retinoic acid was used at 10 µM; Cycloheximide was used at 10 µM to inhibit protein synthesis; and MG132 was used at 10 µM to inhibit proteasomal degradation. Treatment durations ranged from 24 to 72 hours depending on the specific assay. Control cells were treated with the corresponding vehicle (e.g., PBS or DMSO) at equivalent volumes.

### Immunoblotting, immunoprecipitations, and lectin pull-down assay

Cell lysates were prepared using lysis buffer (Thermo Fisher Scientific). Proteins were separated on a 10% SDS-PAGE gel (Bio-Rad), transferred onto PVDF membranes (Millipore), blocked with TBST containing 5% bovine serum albumin (Bio-Rad), and incubated with primary antibodies against O-GlcNAc (Clone RL2, Cat. No. MA1-072, Invitrogen, [1:1000]), GAPDH (Cat. No. GTX627408, GeneTex, [1:5000]), GSK3β (Cat. No. GTX133372, GeneTex, [1:1000]), Phospho-GSK-3β (Ser9) (Cat. No. 9323T, Cell Signaling, [1:1000]), Phospho-c-Myc (Thr58) (Cat. No. 46650T, Cell Signaling, [1:1000]) and MYCN (Cat. No. 84406 S, Cell Signaling, [1:1000]). After incubation with HRP-conjugated secondary antibodies (Cat. No. 111-035-003, Jackson ImmunoResearch, [1:5000]) and HRP-conjugated streptavidin, protein bands were visualized using ECL reagents (GE Healthcare Life Sciences). For lectin pull-down assays, 500 µg of cell lysate proteins, treated or untreated with 0.4 U/mL neuraminidase (New England Biolabs), were incubated with succinylated Wheat Germ Agglutinin (sWGA) lectin-conjugated beads (Vector Laboratories) to enrich for O-GlcNAc-modified proteins. After washing and boiling to elute the proteins, samples were analyzed by SDS-PAGE and western blot.

### Immunofluorescence staining

Cells were seeded at a density of 5 × 10^3^ cells per well in chamber slides (SPL Life Sciences), fixed with 4% PFA, permeabilized with 0.25% Triton X100, blocked in PBS containing 2% bovine serum albumin (Bio-Rad) and applied with primary antibodies to O-GlcNAc (Cat. No. LS‑B2534, LSBio, dilution [1:200]) and NFH (Cat. No. 171 104, Synaptic Systems, dilution [1:500]). Alexa Fluor 488- and 594-conjugated secondary antibodies (Cat. No. A-11008, Cat. No. A-11012, Life Technologies, dilution [1:1000]) were used as secondary antibodies. Isotype control antibodies were used as controls. Cell nuclei were visualized with DAPI (Santa Cruz Biotechnology). Images were captured using a Carl Zeiss LSM880 fluorescent confocal microscope (Carl Zeiss, Jena, Germany) equipped with a 40× objective lens.

### Cell viability assay

Cell viability was assessed using the MTT assay. NB Cells were seeded into 96-well plates at a density of 1.5 × 10³ cells per well and allowed to attach overnight. The following day, cells were treated with Thiamet G (10 µM) or Retinoic acid (10 µM) for 24 and 48 h. Then, 10 µL of a 5 mg/mL MTT solution (3-(4,5-dimethyl-2-thiazolyl)-2,5-diphenyl-2 H-tetrazolium bromide; Sigma) was added to each well for the indicated times and incubated at 37 °C for 3 h, and the MTT formazan crystals were dissolved with 100 µL 10% SDS containing 0.01 N HCl. The resultant optical density was measured spectrophotometrically at the dual wavelengths of 550 and 630 nm using the Epoch microplate reader (BioTek Instruments, Winooski, VT, USA).

### Matrigel invasion assays

Cell invasion assays were evaluated using transwell (Corning, NY, USA) coated with Matrigel (BD Biosciences, CA, USA). Each transwell chamber contained a membrane filter with pore size 8 μm. Each group of NB cells (1 × 10^5^) were seeded into the Matrigel-coated transwell chamber contained with 0.25 mL serum-free DMEM. After incubating for 48 h, the cells were fixed and stained with 0.5% (w/v) crystal violet (Sigma) containing 20% (v/v) methanol. The number of invaded cells was counted in 3 randomly selected fields per well using a Nexcope NE950 inverted light microscope at 100× magnification.

### Transgenic mouse model and in vivo therapeutic experiments

All animal experiments were approved by the Institutional Animal Care and Use Committee (IACUC) of the College of Medicine, National Taiwan University (IACUC approval no. 20210347). Th-MYCN hemizygous mice with a 129 × 1/SvJ genetic background were originally provided by Prof. William Weiss (University of California, San Francisco) via Prof. Akira Nakagawara (Kyushu International External Beam Radiotherapy Center, Japan) [32].

### Animal allocation and inclusion criteria

Inclusion criteria: Mice were included in the study if they developed neuroblastoma as a result of targeted overexpression of the human MYCN gene in the sympathetic nervous system. Approximately 70% of the transgenic mice met this criterion. Exclusion criteria: Mice that did not develop palpable tumors or had incomplete data due to technical issues with tumor size measurements were excluded. Allocation Strategy: Due to the variability in tumor onset and growth rates in this spontaneous model, strict randomization was not employed. Instead, tumor-bearing mice were matched by age and initial tumor volume to ensure baseline comparability between the vehicle control and treatment groups. Sample Size: Sample sizes were determined based on the ~ 70% tumor penetrance rate and pilot experiments, which indicated that a minimum of 6 mice per group is required to detect a significant difference in tumor progression (power > 0.8).

Treatment Protocol: Upon tumor detection (approximately 14–21 days of age), treatment was initiated. Thiamet G (MedChemExpress; HY-12588) was dissolved in PBS at a concentration of 2 mg/ml. Mice received intraperitoneal injections of Thiamet G (20 mg/kg) or vehicle alone every two days (*n* = 6 per group). The treatment duration was set to 30 days, which corresponds to the typical survival window of the vehicle-treated control group in this aggressive model.

#### Assessment endpoints

##### Tumor monitoring

Tumor onset and progression were monitored longitudinally using the VisualSonics VEVO-2100 high-frequency ultrasound system to guide the treatment schedule.

##### Endpoint assessment

At the end of the 30-day treatment period (approximately 45–50 days of age), mice were sacrificed. Representative tumors were photographed to document gross morphology. To quantify tumor burden, tumors were surgically excised, weighed using a precision balance, and their final volume was measured using digital calipers (calculated as 0.5 × L × W × H).

##### Survival analysis

For the survival cohort, the primary endpoint was overall survival. Survival time was defined as the duration from the start of treatment until the mouse died spontaneously or reached the humane endpoint (maximal tumor diameter > 15 mm). Tissue Processing Following excision, tumors were washed and fixed in formalin for subsequent paraffin embedding and immunohistochemical staining.

### Quantitative proteomics analysis

Tumor lysates were digested with trypsin and analyzed using an Orbitrap Fusion Lumos Tribrid mass spectrometer (Thermo Fisher Scientific) coupled to an Ultimate 3000 nanoLC system. Peptides were separated on a C18 column (75 μm × 25 cm) using a 90-min gradient. To optimize the detection of O-GlcNAc-modified peptides, data were acquired using an HCD-product-dependent ETD (HCD-pd-ETD) method, where the detection of HexNAc diagnostic ions (204.0867–138.0545 m/z) triggered subsequent electron transfer dissociation (ETD) scans. Raw data were processed using Proteome Discoverer (version 2.2) and searched against the UniProt mouse reference proteome. Variable modifications included HexNAc (S/T), oxidation (M), and deamidation (N/Q). Label-free quantification (LFQ) was performed to compare protein abundance between groups [33].

### Statistical analysis

The statistical analysis was carried out with SPSS 17.0 (SPSS Inc., Chicago, IL, USA) and GraphPad Prism software. Survival Analysis: Survival probabilities were estimated using the Kaplan-Meier method. For human patient cohorts, differences were analyzed by the log-rank test. For the animal model, differences were assessed using the Gehan-Breslow-Wilcoxon test. Comparison of Groups: For comparisons between two groups, the two-tailed Student’s t-test was used. For comparisons among multiple groups (more than two), one-way analysis of variance (ANOVA) followed by Tukey’s post-hoc test was performed. Data Presentation: All quantitative data are presented as the mean ± standard deviation (SD). Unless otherwise stated, in vitro experiments were performed in triplicate (*n* = 3), and in vivo groups consisted of *n* = 6 mice. A P value < 0.05 was considered statistically significant.

## Results

### Prognostic value and clinicopathological parameters of OGT / OGA mRNA expression in neuroblastoma patients: analysis using public database

**Fig. 1:**
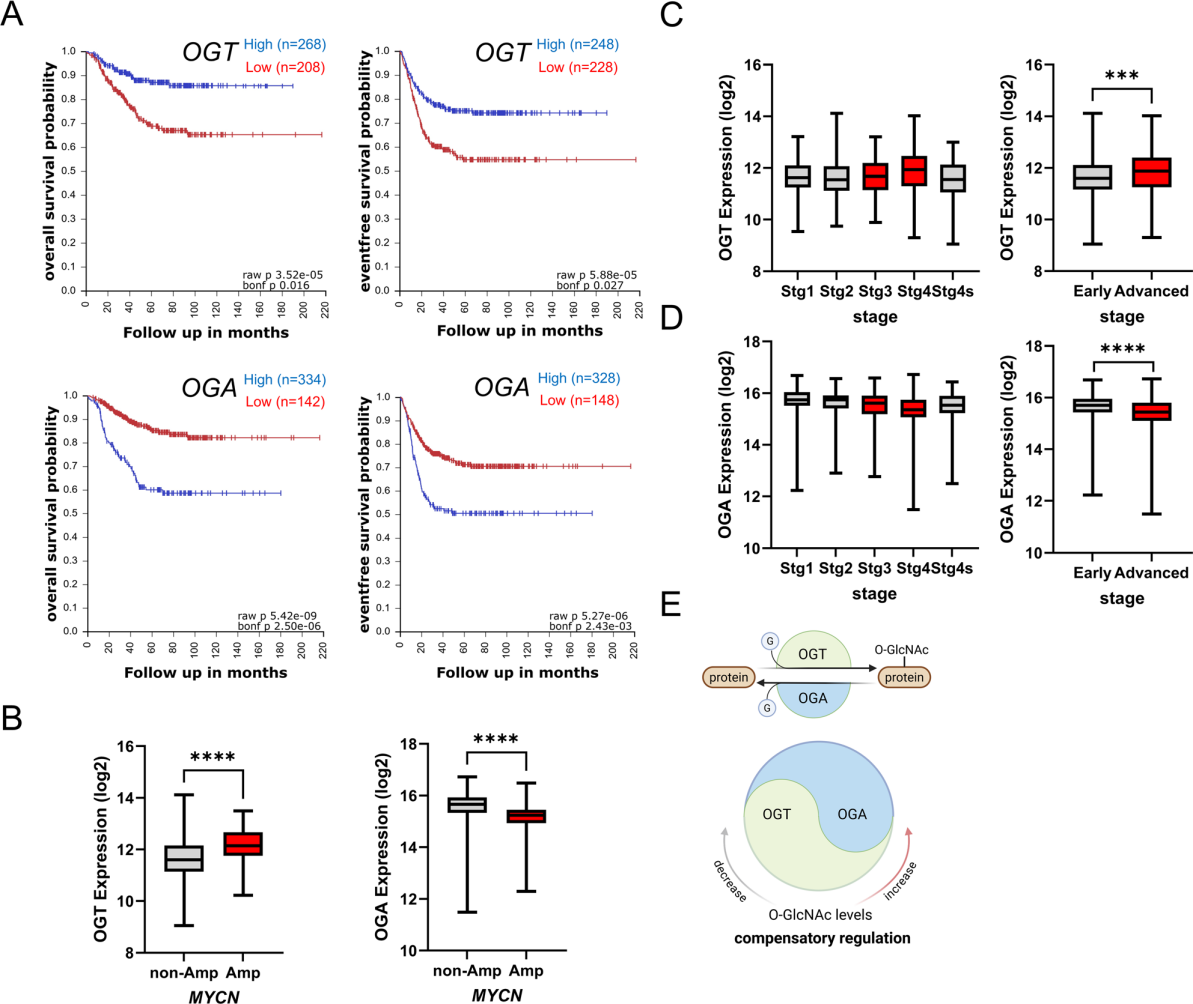
Prognostic significance and clinicopathological correlations of OGT and OGA mRNA expression in neuroblastoma patients. **A** Kaplan–Meier survival analysis of overall survival (OS) and event-free survival (EFS) using the Kocak neuroblastoma dataset (*n* = 649). Patients with high OGT mRNA expression (upper panels) exhibit significantly poorer outcomes, whereas high OGA expression (lower panels) is associated with favorable survival. P-values are indicated in the plots. **B** Box plots showing the expression levels of OGT (left) and OGA (right) in MYCN-non-amplified (non-Amp) versus MYCN-amplified (Amp) tumors. OGT is significantly upregulated, while OGA is downregulated in MYCN-amplified tumors. **C**, **D** Box plots depicting the correlation of (C) OGT and (D) OGA mRNA expression with International Neuroblastoma Staging System (INSS) clinical stages. OGT levels are higher in advanced stages, while OGA levels are lower in advanced stages compared to early stages. **E** Schematic representation of the enzymatic regulation of O-GlcNAcylation. OGT adds O-GlcNAc to proteins, while OGA removes it. The diagram illustrates the dynamic cycling and potential compensatory regulation between these two enzymes. Data are presented as mean ± SD where applicable. Statistical significance was determined using Student’s t-test or log-rank test (***p < 0.001, ****p < 0.0001)

To investigate the prognostic significance of O-GlcNAc cycling enzymes in neuroblastoma (NB), we analyzed OGT and OGA mRNA expression using the Kocak public database (*n* = 649) via the R2: Genomics Analysis and Visualization Platform. Survival analysis revealed a distinct prognostic pattern: NB patients with high OGT mRNA expression exhibited significantly poorer overall survival (*p* = 3.52e-05) and event-free survival (*p* = 5.88e-05) (Fig. 1A, upper panels). Conversely, high expression of OGA, the enzyme responsible for removing O-GlcNAc, was strongly associated with favorable overall and event-free survival (Fig. 1A, lower panels).

We further examined the relationship between these gene expression levels and key clinicopathological risk factors. Box plot analysis demonstrated that OGT expression levels (log2) were significantly elevated in tumors with MYCN amplification compared to non-amplified tumors (Fig. 1B, left). Consistently, OGT expression was significantly higher in patients with advanced clinical stages compared to early stages (Fig. 1C). In contrast, OGA expression showed an inverse pattern, being significantly lower in MYCN-amplified tumors (Fig. 1B, right) and significantly downregulated in advanced clinical stages (Fig. 1D, *p* < 0.0001). Figure 1E summarizes the enzymatic cycle and suggests a potential compensatory expression pattern between OGT and OGA to maintain O-GlcNAc homeostasis in NB. To validate the protein-level expression and localization of O-GlcNAc specifically within tumor cells, we next examined O-GlcNAcylated protein expression using immunohistochemical (IHC) staining in a clinical cohort.

### High O-GlcNAc expression correlates with the differentiation status of neuroblastoma (NB) tumors

**Fig. 2:**
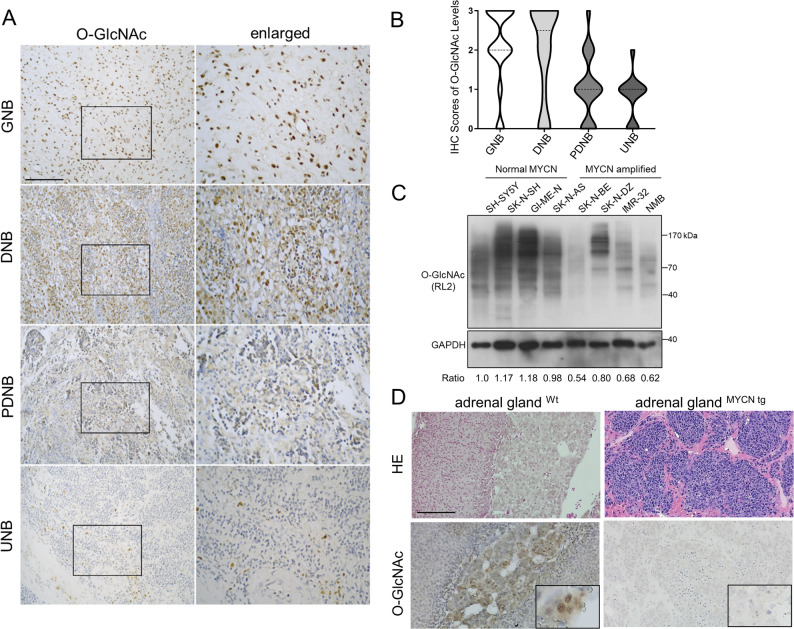
High O-GlcNAc expression correlates with neuroblastoma tumor differentiation. **A** Representative IHC images showing O-GlcNAcylated proteins in neuroblastoma tumors of varying histological types. Brown staining indicates positive cells. Tumors with mature histology (GNB or DNB) exhibit higher O-GlcNAc levels, whereas undifferentiated (UNB) tumors show lower levels. Scale bars: 100 μm; insets show high-magnification views of the marked areas. **B** Violin plots summarizing the percentage of O-GlcNAc expression in 158 neuroblastoma clinical samples. **C** Immunoblot analysis of O-GlcNAc expression in various neuroblastoma cell lines, comparing those with and without MYCN amplification. GAPDH serves as the internal loading control. The numbers below the blots indicate the relative intensity ratio of O-GlcNAc normalized to GAPDH. **D** Representative IHC images of O-GlcNAcylated proteins in adrenal tumors from Th-MYCN transgenic mice. Brown staining indicates positive cells. Scale bars: 100 μm; insets show high-magnification views. NB, neuroblastoma; IHC, immunohistochemistry; GNB, ganglioneuroblastoma; DNB, differentiating neuroblastoma; UNB, undifferentiated neuroblastoma; GAPDH, glyceraldehyde-3-phosphate dehydrogenase

To evaluate the clinical relevance of O-GlcNAcylation, we analyzed O-GlcNAcylated protein expression in a cohort of 158 neuroblastoma (NB) tumors using immunohistochemical (IHC) staining. We observed that positive O-GlcNAc staining was localized primarily to the nuclear compartments of ganglion cells, whereas Schwannian stromal cells showed no immunoreactivity. Notably, tumors with mature histology, such as ganglioneuroblastoma (GNB) and differentiating NB (DNB), exhibited markedly higher levels of O-GlcNAcylated proteins (Fig. 2A). This observation was quantitatively confirmed by violin plots showing significantly elevated IHC scores in differentiated subtypes (Fig. 2B).

In vitro, Western blot analysis of human neuroblastoma cell lines revealed that MYCN-non-amplified lines maintained higher O-GlcNAcylated protein levels than MYCN-amplified lines (Fig. 2C). Consistent with these human data, examination of adrenal glands from Th-MYCN transgenic mice showed that spontaneous MYCN-driven tumors expressed markedly lower levels of O-GlcNAc compared to the robust staining observed in the normal adrenal medulla of wild-type mice (Fig. 2D). Clinically, high O-GlcNAc expression (immunoreactivity 2 + to 3+) was detected in 80 of the 158 tumors (50.6%) and was predominantly associated with differentiated histology and the presence of ganglion cells (Table 1; Supplementary Fig. 1). Collectively, these findings indicate that the intensity and percentage of O-GlcNAc immunostaining are strongly correlated with histological differentiation in neuroblastoma.

**Table 1:** O-GlcNAc expression and clinicopathologic and biologic characteristics of NB

	Cases	Positive O-GlcNAc expression (%)	*P* value
Age at diganosis			
≤ 1.5 year	56	38 (67.9)	0.002
> 1.5 year	102	42 (41.2)	
Sex			
Male	96	41 (42.7)	0.015
Female	62	39 (62.9)	
Clinical stage			
1, 2, 4 S	52	33 (63.5)	0.028
3, 4	106	47 (44.3)	
Primary tumor site			
Adrenal	105	49 (46.7)	0.180
Extra-adrenal	53	31 (58.5)	
Tumor histology			
Undifferentiated^a^	109	49 (45.0)	0.040
Differentiated^b^	49	31 (63.3)	
MYCN			
Amplified	37	9 (24.3)	< 0.001
Non-amplified	121	71 (58.7)	

^a﻿^including undifferentiated and poorly differentiated NB

^b^including differentiating NB and ganglioneuroblastoma

### Elevated O-GlcNAc levels independently predict favorable neuroblastoma prognosis

**Fig. 3:**
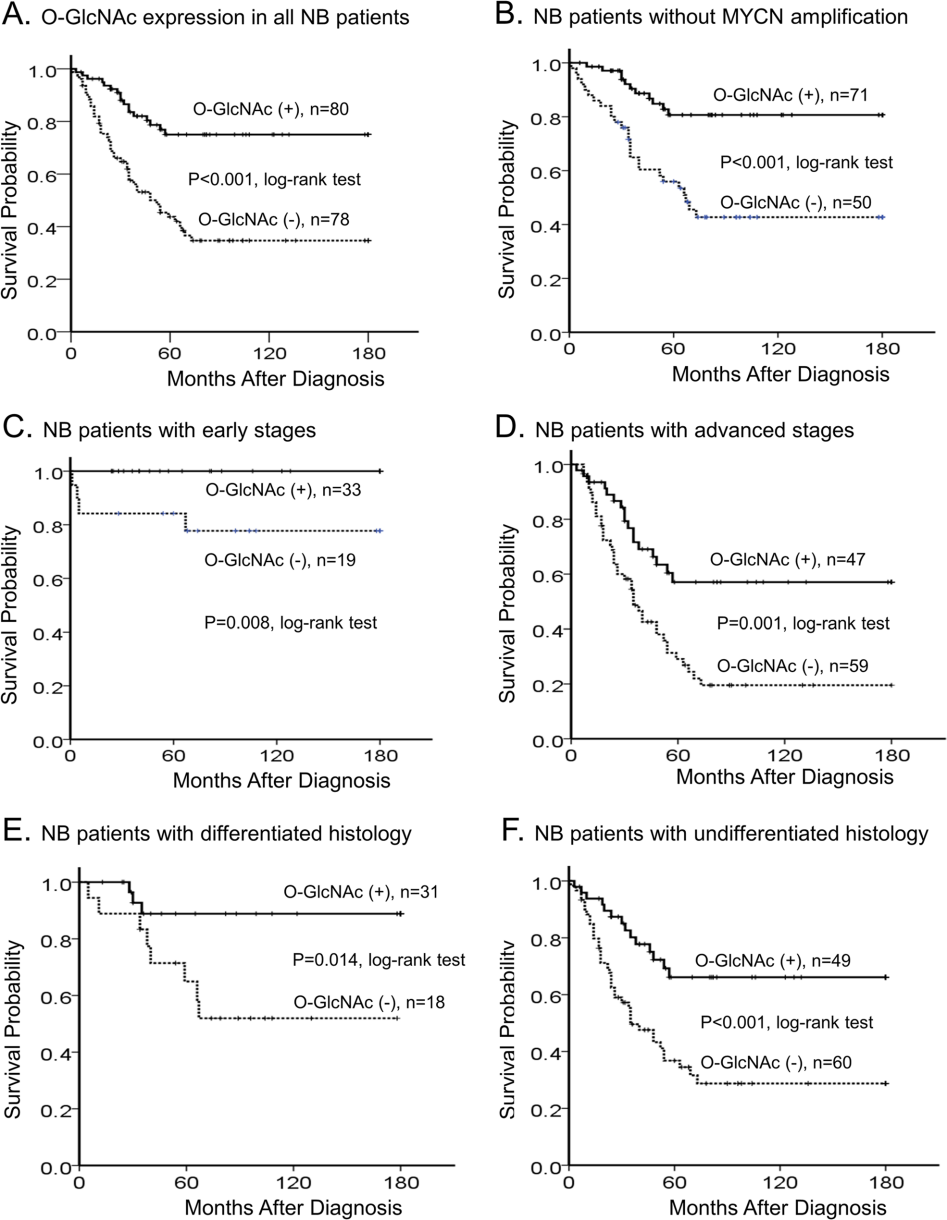
Elevated O-GlcNAc levels independently predict favorable neuroblastoma prognosis. **A** Kaplan–Meier overall survival analysis based on O-GlcNAc expression in 158 neuroblastoma patients (*p* < 0.001, log-rank test). **B-F** Kaplan–Meier survival analyses based on O-GlcNAc expression in subgroups of patients with MYCN non-amplified (*p* < 0.001), early stages (*p* = 0.008), advanced stages (*p* = 0.001), differentiated histology (*p* = 0.014), and undifferentiated histology (*p* < 0.001), respectively (log-rank tests)

To evaluate the prognostic significance of O-GlcNAcylation, we performed survival analysis on our cohort. Kaplan-Meier analysis revealed that patients with high O-GlcNAcylated protein expression had significantly better overall survival rates compared to those with low expression (immunoreactivity “–” or “1+”) (Fig. 3A; *p* < 0.001, log-rank test). The association between O-GlcNAc expression and clinicopathologic variables is summarized in Table 1. Of the 158 NB tumors, 37 (23.4%) harbored MYCN amplification. When tumors were categorized by O-GlcNAc expression levels (low: “–” or “1+”; high: “2+” or “3+”), significant correlations were observed (Table 1; Supplementary Fig. 1). Notably, high O-GlcNAc expression was significantly associated with differentiated tumor histology and early clinical stages (INSS stages 1, 2, and 4 S) (*p* < 0.05, Table 1). Univariate analysis identified low O-GlcNAc expression, age at diagnosis > 1.5 years, advanced clinical stages (INSS stages 3 and 4), MYCN amplification, and undifferentiated histology as strong predictors of poor survival (Table 2). Importantly, multivariate analysis confirmed that clinical stage, MYCN amplification, undifferentiated histology, and low O-GlcNAc expression remained independent prognostic factors for poor outcome (Table 2). Subgroup analyses further demonstrated the robust prognostic value of O-GlcNAc. High O-GlcNAc expression consistently predicted better survival outcomes in patients with MYCN non-amplified tumors (Fig. 3B, *p* < 0.001), early-stage disease (Fig. 3C, *p* = 0.008), advanced-stage disease (Fig. 3D, *p* = 0.001), differentiated histology (Fig. 3E, *p* = 0.014), and undifferentiated histology (Fig. 3F, *p* < 0.001).

**Table 2:** Clinicopathologic and biologic factors affecting survival rate

Variable	Univariate analysis			Multivariate analysis		
	RR	95% CI	*P* value	RR	95% CI	*P* value
Age at diagnosis:						
> 1.5 year versus		2.232–8.513				
≤ 1.5 year	4.359		< 0.001	1.944	0.768–4.922	0.160
Clinical stage:						
advanced (3, 4)		4.699–35.561			1.513–15.969	
versus early (1, 2, 4 S)	12.926		< 0.001	4.916		0.008
MYCN:						
amplified versus non-amplified	3.959	2.464–6.362	< 0.001	1.720	1.002–2.952	0.049
O-GlcNAc expression:						
negative versus positive	3.468	1.986–6.005	< 0.001	1.982	1.083–3.627	0.026
Tumor histology:						
undifferentiated versus differentiated	2.356	1.334–4.161	0.003	2.192	1.114–4.315	0.023

*Abbreviations*: *RR* Risk ratio, *95% CI* 95% Confidence interval

### Pharmacological inhibition of OGA activity with Thiamet G ameliorates neuroblastoma malignant phenotypes, comparable to retinoic acid

**Fig. 4:**
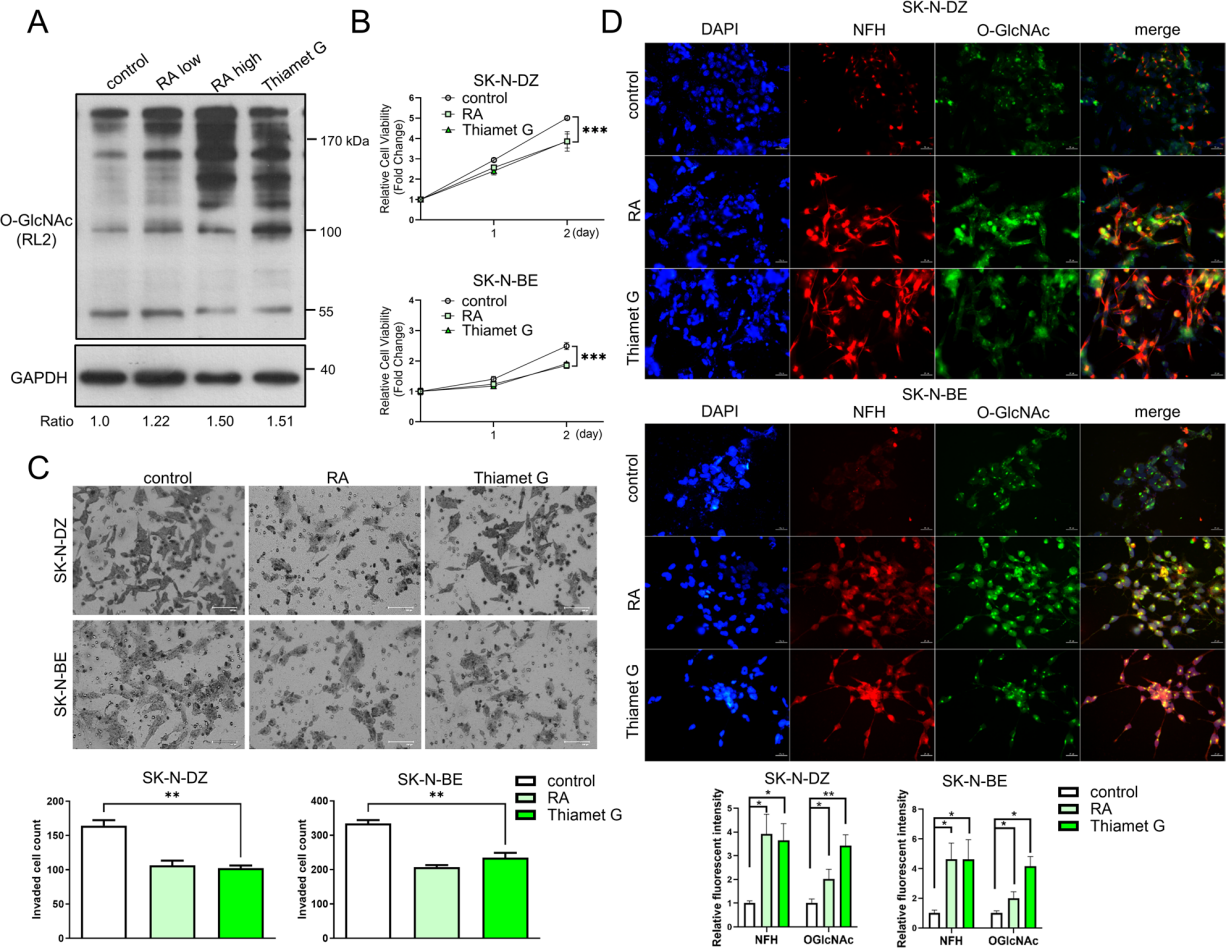
Thiamet G reduces neuroblastoma malignancy similarly to retinoic acid. SK-N-BE and SK-N-DZ cells were treated with Thiamet G (10 µM) or retinoic acid (RA; 10 µM) for the indicated times. Control cells were treated with vehicle alone. **A** Western blot analysis of O-GlcNAc expression in SK-N-BE cells treated for 48 h. GAPDH was used as the internal loading control. The numbers below the blots indicate the relative intensity ratio of O-GlcNAc normalized to GAPDH. **B** MTT assay analysis showing significantly decreased cell proliferation in Thiamet G- and RA-treated groups compared to the control. Data are presented as the mean cell viability fold change ± SD relative to the control at day 0 (n = 3; ***p < 0.001). **C** Representative images from the transwell invasion assay. The bar graph represents the mean number of invaded cells ± SD (n = 3; *p* < 0.01). Scale bar: 100 μm. **D** Neurite outgrowth analysis with representative immunofluorescence staining of NFH (red) and O-GlcNAc (green) in cells treated for 3 days. Images were acquired by confocal microscopy. Nuclei were stained with DAPI (blue). Quantification of fluorescence intensity is shown in the right panel. Data represent mean ± SD (n = 3; *p < 0.01). Scale bar: 100 μm. RA, retinoic acid; GAPDH, glyceraldehyde-3-phosphate dehydrogenase; MTT, 3-(4,5-dimethylthiazol-2-yl)-2,5-diphenyltetrazolium bromide; SD, standard deviation; NFH, neurofilament heavy chain; DAPI, 4′,6-diamidino-2-phenylindole

Consistent with our clinical findings, basal O-GlcNAc expression varies across different NB cell lines, with the highest levels observed in MYCN-non-amplified cells. To investigate whether restoring O-GlcNAc levels could reverse the malignant phenotype, we treated MYCN-amplified cells (SK-N-BE and SK-N-DZ) with Thiamet G, a potent OGA inhibitor, and compared its efficacy to that of Retinoic Acid (RA), a standard differentiation therapy agent. Western blot analysis confirmed a significant upregulation of O-GlcNAcylated proteins in NB cells treated with either RA or Thiamet G compared to controls (Fig. 4A). Functionally, Thiamet G treatment markedly suppressed cell proliferation, as demonstrated by the MTT assay (Fig. 4B). In Matrigel invasion assays, Thiamet G significantly inhibited the invasive potential of NB cells, reducing invasion to approximately 67% of control levels (Fig. 4C). Furthermore, confocal microscopy revealed that Thiamet G-induced O-GlcNAc accumulation promoted morphological differentiation, characterized by extensive neurite outgrowth—a hallmark of neuronal differentiation—as evidenced by NFH staining (Fig. 4D). Quantitative analysis confirmed a significant increase in neurite length (or differentiation rate) in Thiamet G-treated cells compared to controls (*p* < 0.01). Notably, the phenotypic improvements induced by Thiamet G were comparable to those observed with RA treatment.

### The effect of Thiamet G-mediated OGA inactivation in *Th-MYCN* transgenic mice

**Fig. 5:**
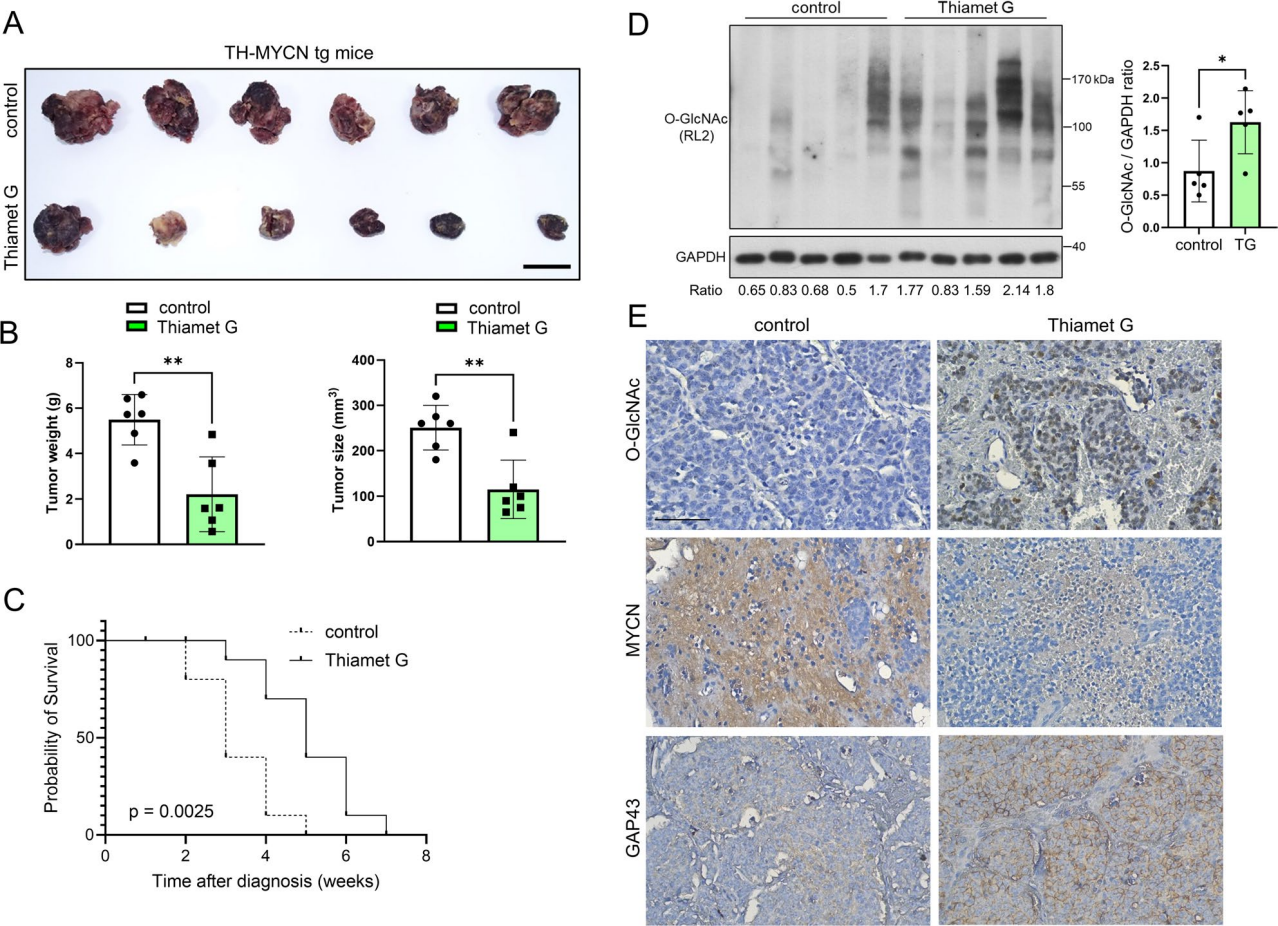
Effect of Thiamet G in Th-MYCN Mice. **A** Representative images of primary tumors from control and Thiamet G-treated mice (n = 6 per group). Scale bars: 1 cm. **B** Scatter plots with bars showing tumor weights and sizes for mice sacrificed at day 45. Data are presented as mean ± SD (p < 0.01). **C** Kaplan-Meier survival analysis of mice in control and Thiamet G-treated groups (n = 10 per group). Thiamet G treatment significantly prolonged survival compared to the control group (p < 0.001, log-rank test), resulting in a 40% increase in median survival time. **D** Western blot analysis demonstrating stable O-GlcNAc overexpression in Thiamet G-treated mice. Tumor samples were obtained from the tumor growth cohort (described in A and B). GAPDH was used as the internal loading control. The numbers below the blots indicate the relative intensity ratio of O-GlcNAc normalized to GAPDH. The bar graph (right panel) represents the densitometric quantification of O-GlcNAc levels normalized to GAPDH (mean ± SD; p < 0.01). **E** Representative immunohistochemical images of O-GlcNAc, MYCN, and GAP43 staining in tumors from control and Thiamet G-treated mice (from the same cohort as in A). Brown staining indicates positive cells. Quantitative analysis of the IHC staining is shown in Supplementary Fig. 2. Scale bars: 100 μm. SD, standard deviation; GAPDH, glyceraldehyde-3-phosphate dehydrogenase; GAP43, growth associated protein 43; IHC, immunohistochemistry

Our clinical analysis revealed that elevated O-GlcNAc levels independently predict a favorable prognosis in neuroblastoma, particularly showing a strong inverse correlation with MYCN amplification. To validate these findings in vivo, we investigated the therapeutic efficacy of Thiamet G in the *Th-MYCN* transgenic mouse model, which spontaneously develops *MYCN*-driven neuroblastoma. High-resolution small-animal ultrasound imaging confirmed a significant reduction in tumor growth rate in Thiamet G-treated mice compared to controls (Fig. 5A–B). Furthermore, Thiamet G treatment significantly prolonged the survival of *Th-MYCN* mice. The median survival in the treatment group was 7 weeks, compared to 5 weeks in the control group, representing a 40% extension in lifespan (Fig. 5C; *p* < 0.001, log-rank test). Mechanistically, Western blot analysis of tumor tissues demonstrated that Thiamet G treatment effectively increased global O-GlcNAcylation levels (Fig. 5D). Immunohistochemical (IHC) staining further confirmed that this O-GlcNAc accumulation was predominantly localized to the nucleus of tumor cells (Fig. 5E). Consistent with our in vitro findings, Thiamet G-treated tumors exhibited reduced MYCN expression and increased expression of the neuronal differentiation marker GAP43 (Fig. 5E). Quantitative analysis of the IHC staining further supported these observations (Supplementary Fig. 2).

### Thiamet-G-induced O-GlcNAc accumulation promotes MYCN degradation via the GSK3β-proteasome pathway

**Fig. 6:**
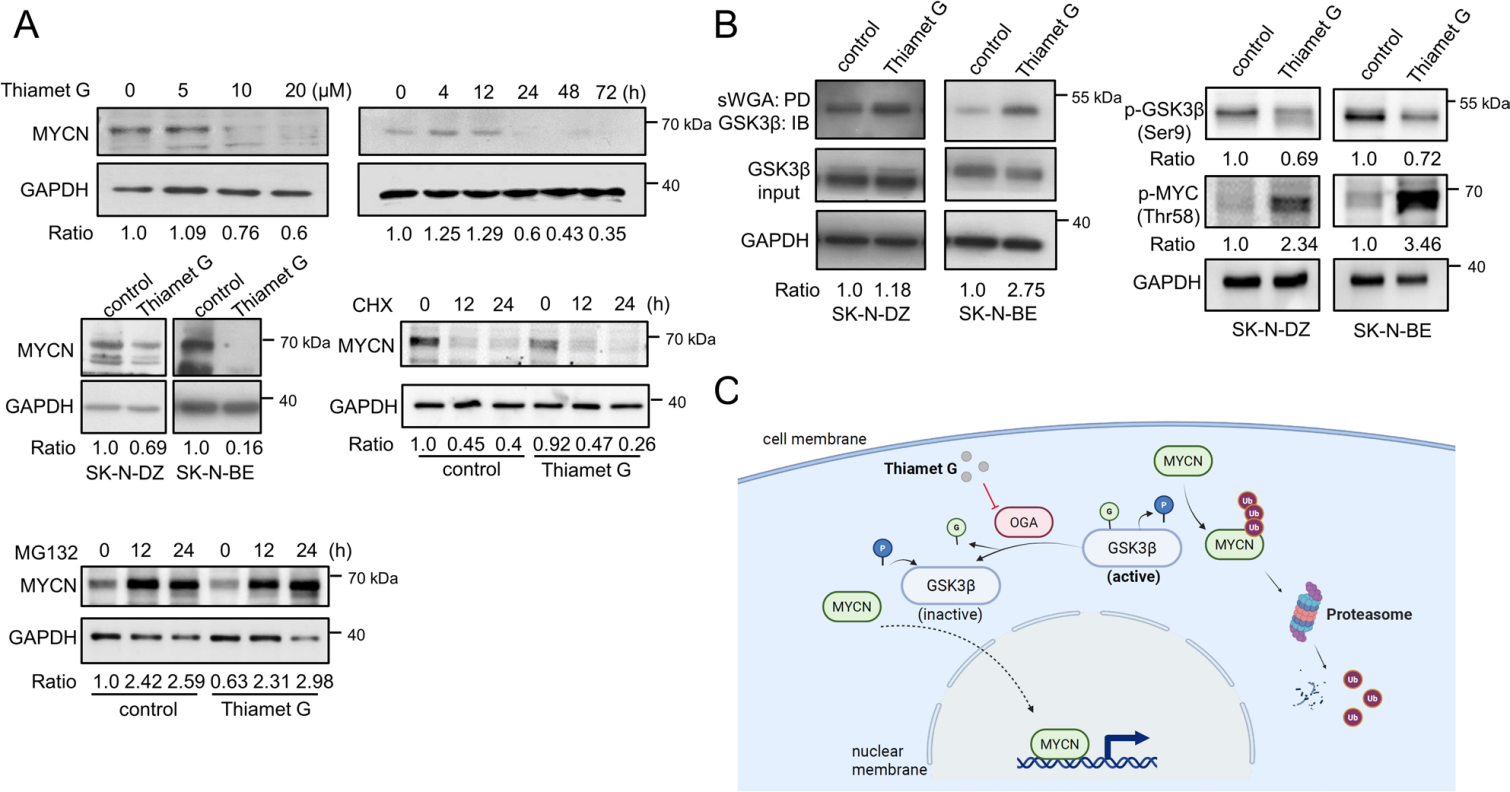
Thiamet-G-induced O-GlcNAc accumulation promotes MYCN degradation via the GSK3β-proteasome pathway. **A** Upper panel: Western blots showing dose- and time-dependent MYCN expression in Thiamet G-treated SK-N-DZ cells. GAPDH serves as the internal loading control. Lower panel: Western blot analysis of MYCN stability in SK-N-BE cells treated with Thiamet G in combination with cycloheximide (CHX) and the proteasome inhibitor MG132 (10 µM each). The restoration of MYCN levels by MG132 indicates proteasome-dependent degradation. The numbers below the blots indicate the relative intensity ratio of O-GlcNAc normalized to GAPDH. **B** Left panel: Total lysates from SK-N-DZ and SK-N-BE cells treated with Thiamet G were pulled down using sWGA agarose beads and immunoblotted with GSK3β antibodies to detect O-GlcNAcylated GSK3β. The numbers below the blots indicate the relative intensity ratio of sWGA pulled down GSK3β normalized to input GSK3β. Right panel: Western blot analysis of phospho-GSK3β (Ser9) and phospho-MYCN (Thr58) expression levels. The reduction in inhibitory p-GSK3β (Ser9) levels was accompanied by a concurrent increase in p-MYCN levels. The numbers below the blots indicate the relative intensity ratio of phospho-GSK3β (Ser9) and phospho-MYCN (Thr58) normalized to GAPDH. **C** Schematic illustration of the proposed mechanism: accumulated O-GlcNAcylation activates GSK3β (by reducing inhibitory Ser9 phosphorylation), leading to MYCN phosphorylation (Thr58) and subsequent proteasomal degradation. GAPDH, glyceraldehyde-3-phosphate dehydrogenase; CHX, cycloheximide; sWGA, succinylated wheat germ agglutinin; GSK3β, glycogen synthase kinase-3 beta; OGA, O-GlcNAcase; p-MYCN, phosphorylated MYCN

To investigate whether manipulating O-GlcNAc levels directly regulates MYCN protein stability, we treated NB cells with Thiamet G. Western blot analysis revealed that MYCN protein expression decreased in a time- and dose-dependent manner in both SK-N-DZ and SK-N-BE cells (Fig. 6A, upper panel). To confirm the mechanism of degradation, we treated cells with the protein synthesis inhibitor cycloheximide (CHX) and the proteasome inhibitor MG132. As shown in Fig. 6A (lower panel), Thiamet G treatment accelerated the decay of MYCN protein compared to controls. Crucially, co-treatment with MG132 effectively reversed the Thiamet G-induced reduction in MYCN levels, confirming that O-GlcNAc accumulation promotes MYCN turnover primarily through the ubiquitination-dependent proteasomal pathway. To elucidate the upstream molecular mechanism, we performed sWGA lectin pull-down assays. Thiamet G treatment increased the amount of GSK3β precipitated by sWGA beads, indicating direct O-GlcNAc modification of GSK3β (Fig. 6B, left panel). Concurrently, Western blot analysis revealed a significant reduction in inhibitory phospho-GSK3β (Ser9) levels in Thiamet G-treated cells. Importantly, this reduction in GSK3β phosphorylation was accompanied by the appearance of phosphorylated MYCN (p-MYCN) (Fig. 6B, right panel). This inverse correlation suggests that O-GlcNAc-mediated activation of GSK3β leads to the direct phosphorylation of MYCN at Thr58, thereby marking it for proteasomal degradation. A schematic model of this regulation is illustrated in Fig. 6C, depicting how O-GlcNAc accumulation activates GSK3β, leading to MYCN destabilization. Finally, to assess the broader metabolic impact of this regulation, functional enrichment analysis of global protein expression revealed that O-GlcNAc accumulation modulates several key pathways, including glycolysis, the pentose phosphate pathway (PPP), the TCA cycle, glutamine metabolism, and RNA processing (Supplementary Fig. 3).

## Discussion

In this study, we analyzed tumor samples from a large cohort of NB patients and demonstrated that O-GlcNAcylated proteins were significantly overexpressed in ganglion cells and well-differentiated tumors. High levels of O-GlcNAc were strongly associated with favorable clinicopathologic features, including age, early disease stage, differentiated histology, and lack of MYCN amplification. Importantly, high O-GlcNAc expression emerged as an independent prognostic factor for superior survival outcomes, providing complementary prognostic value to established markers such as age, tumor differentiation, and MYCN status. Mechanistically, we found that increasing O-GlcNAc levels reduced the malignant properties of NB cells in vitro and suppressed MYCN-driven tumorigenesis in vivo. Our findings reveal that O-GlcNAc acts as a key negative regulator of MYCN stability. Specifically, inhibiting OGA activity with Thiamet G reduced cell proliferation and invasion while enhancing neuronal differentiation and neurite outgrowth. Furthermore, we discovered that high O-GlcNAc levels promote GSK3β activation, thereby accelerating MYCN degradation via the proteasome. To our knowledge, this is the first study to highlight the critical role of O-GlcNAcylation in regulating MYCN signaling in NB, offering new insights into potential therapeutic strategies.

While many reports document alterations in bulk O-GlcNAcylation in various human tumors [34, 35], most rely primarily on transcriptional analysis of OGT and OGA. However, few studies have investigated the actual protein expression patterns of O-GlcNAc in clinical tissues. The intracellular balance of O-GlcNAc is maintained by the interplay between OGT and OGA. Analysis of public datasets (Kocak) suggested that NB patients with high OGT and low OGA mRNA expression had poor outcomes, implying a correlation with aggressive phenotypes. In stark contrast, our protein-level analysis in a larger patient cohort demonstrated that high O-GlcNAc levels predict a better prognosis and inversely correlate with MYCN amplification. This discrepancy likely arises because transcriptomic data cannot capture post-translational enzymatic feedback loops, and bulk RNA sequencing aggregates signals from the entire tumor microenvironment, including stromal cells. This underscores the necessity of using immunohistochemical (IHC) staining to visualize actual O-GlcNAc levels specifically within tumor cells. Our approach provides a more accurate representation of the functional O-GlcNAc landscape in NB, distinct from the transcriptional noise of the stroma.

Cancer cells typically undergo metabolic reprogramming, such as the Warburg effect, to support rapid proliferation [36]. High O-GlcNAc expression has been associated with this metabolic switch and tumor aggressiveness in various epithelial cancers [37–39]. Crucially, however, our findings reveal a diametrically opposite role for O-GlcNAc in neuroblastoma. While OGA inhibition fuels malignancy in breast, prostate, and colorectal cancers, it effectively suppresses it in neuroblastoma. We propose that this discrepancy reflects the fundamental biological divergence between neural and epithelial lineages. Unlike in epithelial tissues where hyper-O-GlcNAcylation often signals metabolic stress, high levels of O-GlcNAc are intrinsic to the healthy brain and essential for neuronal survival, synaptic plasticity, and protection against neurodegeneration [40–43]. This explains our observation that high O-GlcNAc expression is enriched in well-differentiated neuroblastomas, which resemble mature sympathetic neurons. Indeed, our proteomic analysis indicated that O-GlcNAcylated proteins are involved in key metabolic pathways, including the TCA cycle and glutamine metabolism. Pharmacological inhibition of OGA (Thiamet G) does not act as an oncogenic stimulus here; instead, it appears to recapitulate the physiological O-GlcNAc landscape of a differentiated neuron. Consistent with this, Thiamet G promoted neurite outgrowth similar to retinoic acid, aligning with studies showing that O-GlcNAcylation can shift metabolism from glycolysis to oxidative phosphorylation (OXPHOS) to support differentiation [44, 45].

MYCN amplification is a major driver of poor prognosis in neuroblastoma [46]. The stability of MYCN protein is tightly regulated by the ubiquitin-proteasome system [47, 48]. A critical step in this process is the phosphorylation of MYCN at Thr58 by GSK3β, which marks it for degradation by E3 ligases [49]. GSK3β activity is strictly controlled, primarily through inhibitory phosphorylation at Ser9 [50, 51]. Our study provides a novel mechanism linking metabolic signaling to this degradation pathway. We demonstrated that Thiamet G-induced O-GlcNAc accumulation leads to a significant reduction in inhibitory p-GSK3β (Ser9) levels. Mechanistically, our sWGA pull-down results suggest that direct O-GlcNAcylation of GSK3β prevents its inhibitory phosphorylation, thereby keeping it in an active state. This activated GSK3β then phosphorylates MYCN, targeting it for proteasomal degradation, as confirmed by our MG132 rescue experiments. Thus, O-GlcNAcylation serves as a “metabolic brake” on MYCN oncogenic signaling by sustaining GSK3β activity.

In conclusion, our study establishes that high O-GlcNAc expression is a robust marker of favorable prognosis in neuroblastoma. This protective effect is mediated by the stabilization of neuronal differentiation programs and the direct destabilization of the oncoprotein MYCN via the O-GlcNAc-GSK3β axis. These findings challenge the dogma that high O-GlcNAc is universally oncogenic and propose that targeting O-GlcNAc regulation could be a promising therapeutic strategy for differentiating MYCN-driven neuroblastomas.

## Supplementary Information

Supplementary Material 1. Supplementary Fig. 1: Correlation of O-GlcNAc positivity with clinical features in neuroblastoma patients. Bar graph showing the percentage of high O-GlcNAc expression in patient subgroups stratified by MYCN amplification status (Non-amplified vs. Amplified) and tumor histology (Differentiated vs. Undifferentiated). Statistical significance was determined using Pearson's chi-square test (***P < 0.001, *P < 0.05). Data are derived from Table 1.

Supplementary Material 2. Supplementary Fig. 2: Quantitative analysis of immunohistochemical staining in Th-MYCN transgenic mice tumors.Quantification of (A) O-GlcNAc, (B) MYCN, and (C) GAP43 expression levels in tumor tissues from control and Thiamet G-treated Th-MYCN mice (corresponding to the representative images in Fig. 5E). Data are presented as the mean percentage of positive cells (or H-score) ± SD (n = 6 per group). Thiamet G treatment significantly increased O-GlcNAc and GAP43 levels while reducing MYCN expression compared to the control group. **p < 0.01 (Student’s t-test).

Supplementary Material 3: Supplementary Fig. 3: Functional enrichment analysis of O-GlcNAc-regulated pathways in neuroblastoma. Pathway enrichment analysis of the global proteomic dataset from Thiamet G-treated TH-MYCN tumors compared to controls. The analysis reveals that Thiamet G-induced O-GlcNAc accumulation significantly impacts key metabolic pathways, including glycolysis, the pentose phosphate pathway (PPP), the tricarboxylic acid (TCA) cycle, and glutamine metabolism, as well as RNA processing.

Supplementary Material 4.

Supplementary Material 5.

Supplementary Material 6.

Supplementary Material 7.

Supplementary Material 8.

## Data Availability

The data generated in this study are available upon request from the corresponding author.
