# Supplementary figures and images for: O-GlcNAcylation expression predicts a favorable prognosis and mitigates malignant phenotypes via MYCN suppression in neuroblastoma
Source: Mol Cell Pediatr. 2026 Feb 10;13:5. doi: 10.1186/s40348-026-00218-3 (PMC12891278; doi:10.1186/s40348-026-00218-3)

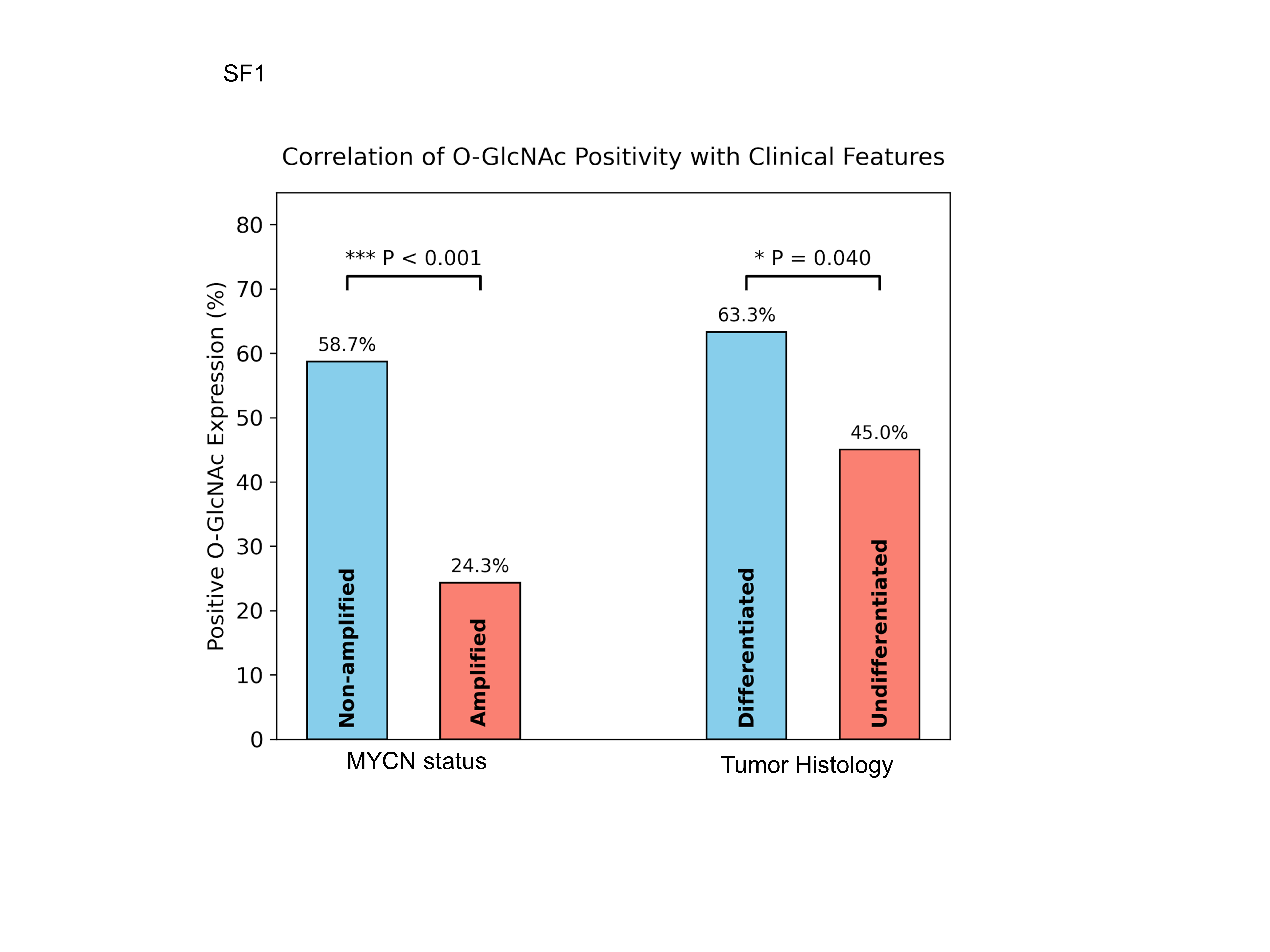

Supplement: Supplementary file 1 — Supplementary Material 1. Supplementary Fig. 1: Correlation of O-GlcNAc positivity with clinical features in neuroblastoma patients. Bar graph showing the percentage of high O-GlcNAc expression in patient subgroups stratified by MYCN amplification status (Non-amplified vs. Amplified) and tumor histology (Differentiated vs. Undifferentiated). Statistical significance was determined using Pearson's chi-square test (***P < 0.001, *P < 0.05). Data are derived from Table 1. [file 40348_2026_218_MOESM1_ESM.tif]

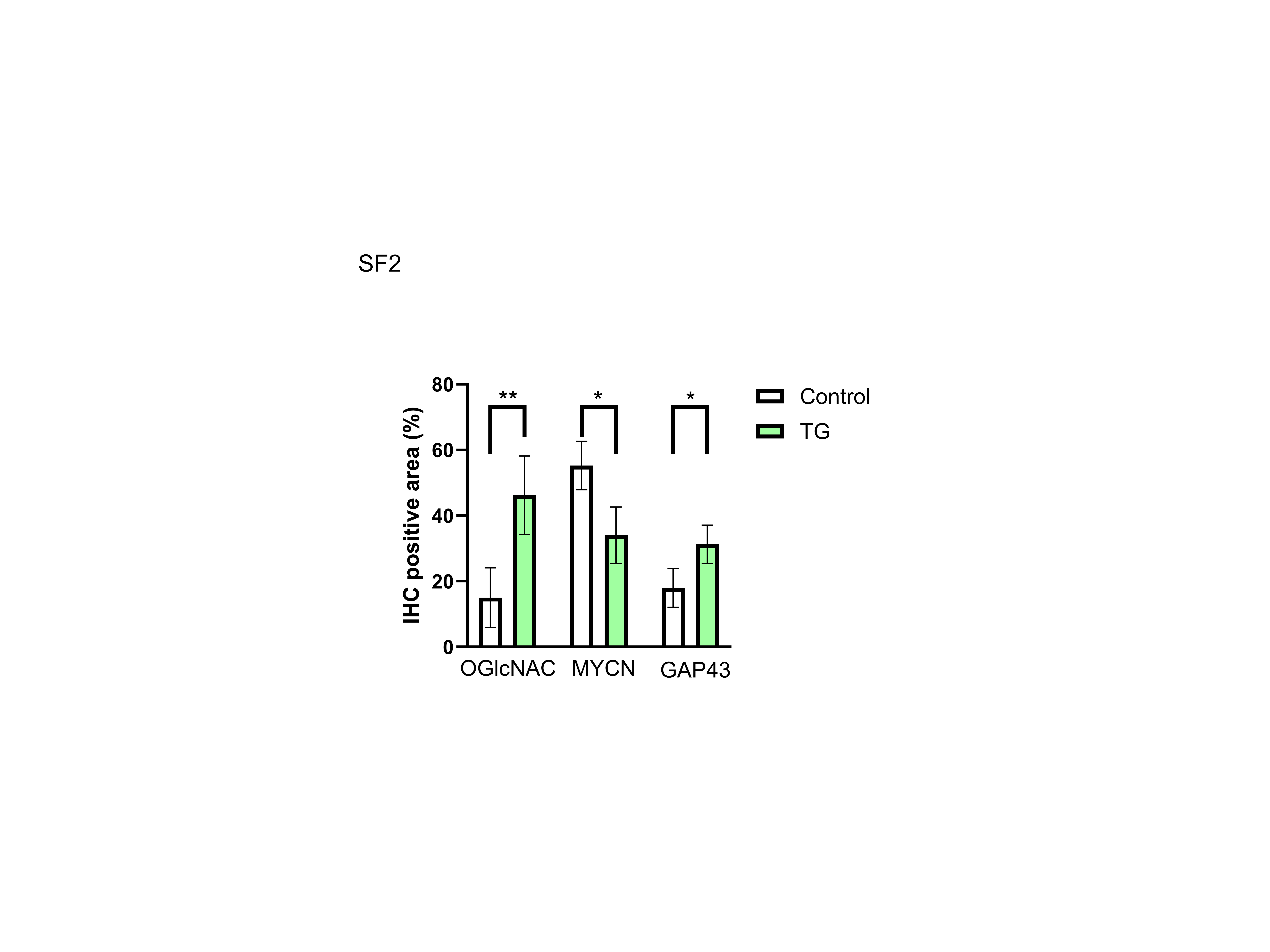

Supplement: Supplementary file 2 — Supplementary Material 2. Supplementary Fig. 2: Quantitative analysis of immunohistochemical staining in Th-MYCN transgenic mice tumors.Quantification of (A) O-GlcNAc, (B) MYCN, and (C) GAP43 expression levels in tumor tissues from control and Thiamet G-treated Th-MYCN mice (corresponding to the representative images in Fig. 5E). Data are presented as the mean percentage of positive cells (or H-score) ± SD (n = 6 per group). Thiamet G treatment significantly increased O-GlcNAc and GAP43 levels while reducing MYCN expression compared to the control group. **p < 0.01 (Student’s t-test). [file 40348_2026_218_MOESM2_ESM.tif]

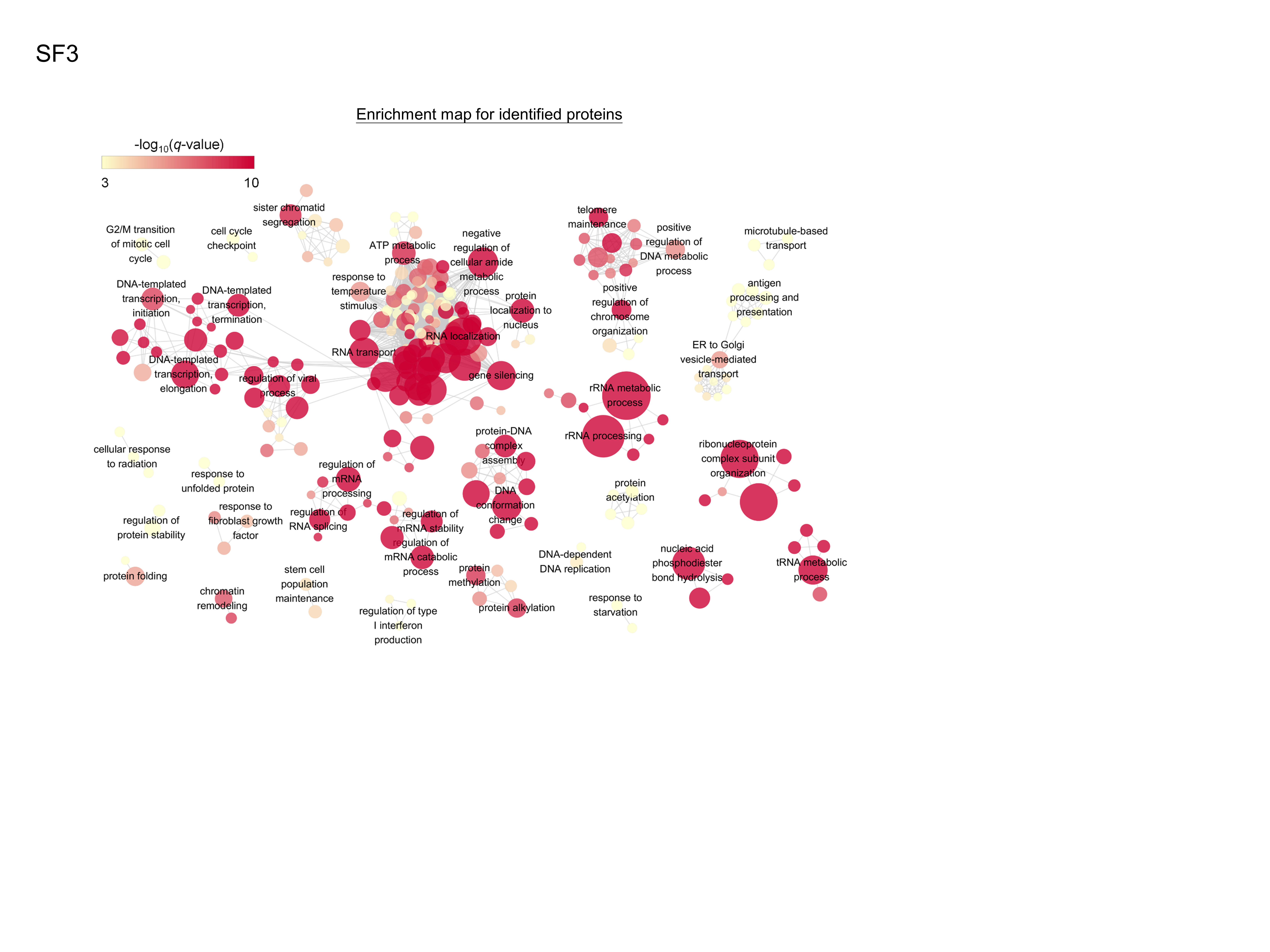

Supplement: Supplementary file 3 — Supplementary Material 3: Supplementary Fig. 3: Functional enrichment analysis of O-GlcNAc-regulated pathways in neuroblastoma. Pathway enrichment analysis of the global proteomic dataset from Thiamet G-treated TH-MYCN tumors compared to controls. The analysis reveals that Thiamet G-induced O-GlcNAc accumulation significantly impacts key metabolic pathways, including glycolysis, the pentose phosphate pathway (PPP), the tricarboxylic acid (TCA) cycle, and glutamine metabolism, as well as RNA processing. [file 40348_2026_218_MOESM3_ESM.tif]

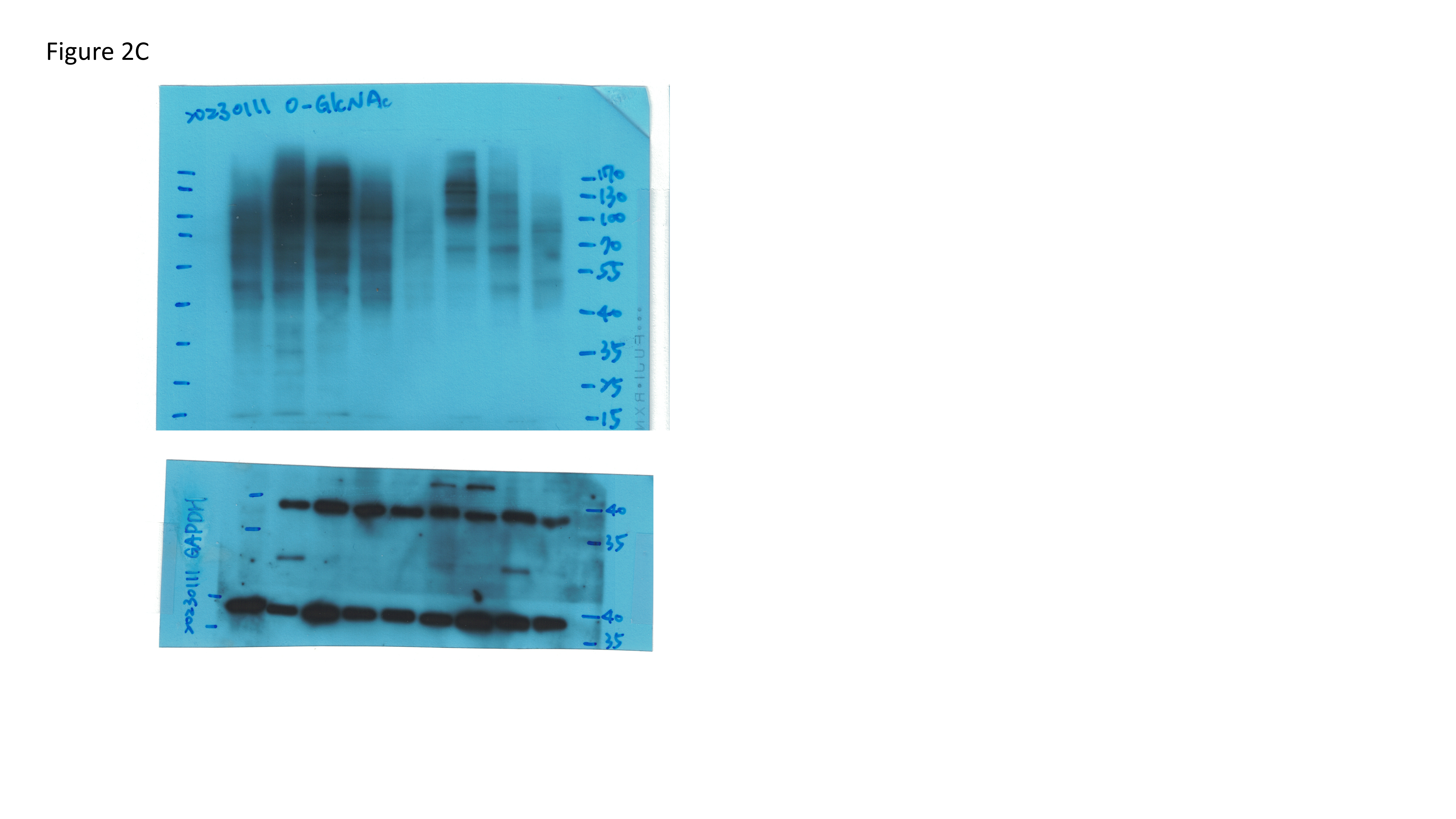

Supplement: Supplementary file 4 — Supplementary Material 4. [file 40348_2026_218_MOESM4_ESM.png]

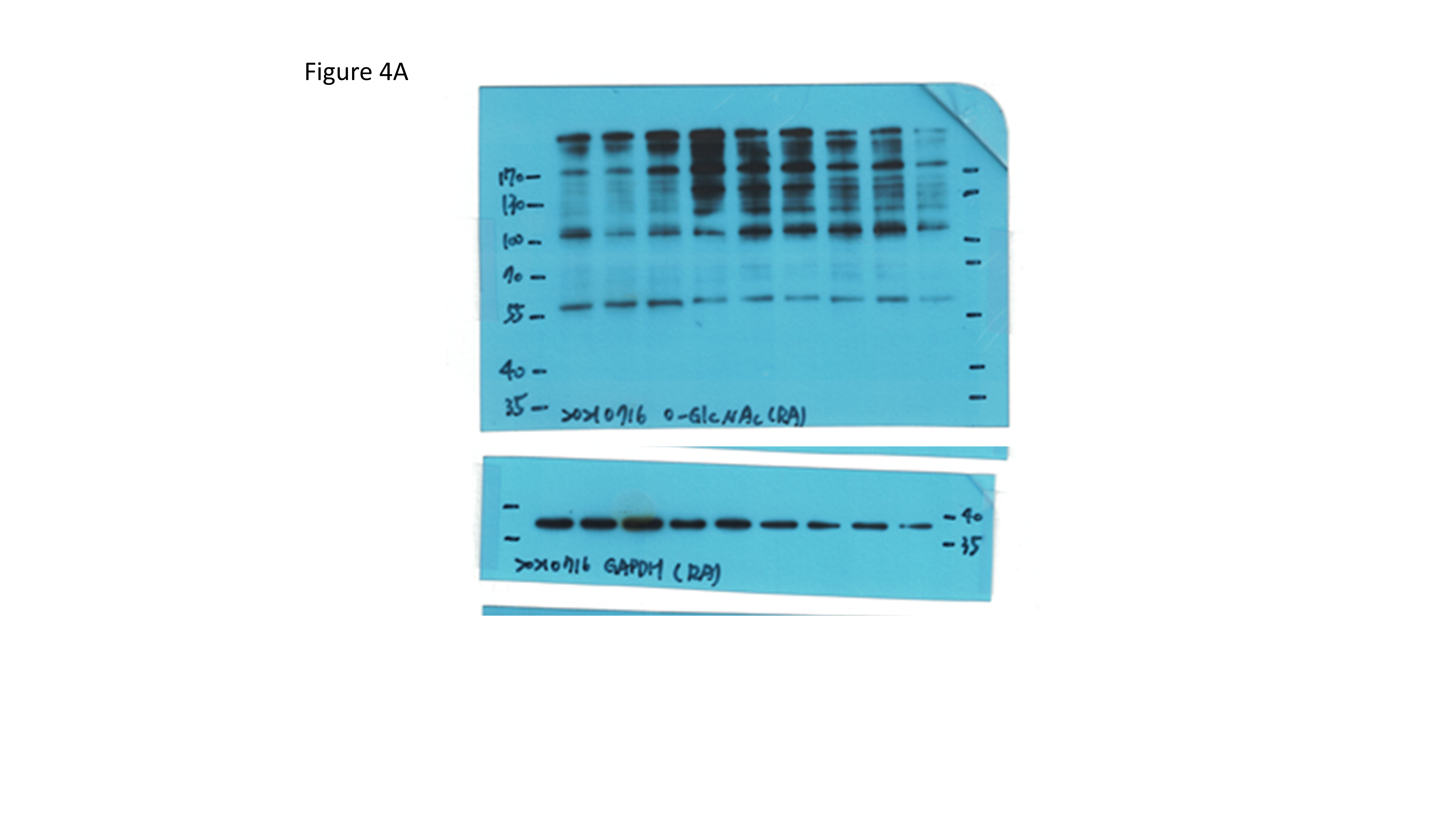

Supplement: Supplementary file 5 — Supplementary Material 5. [file 40348_2026_218_MOESM5_ESM.png]

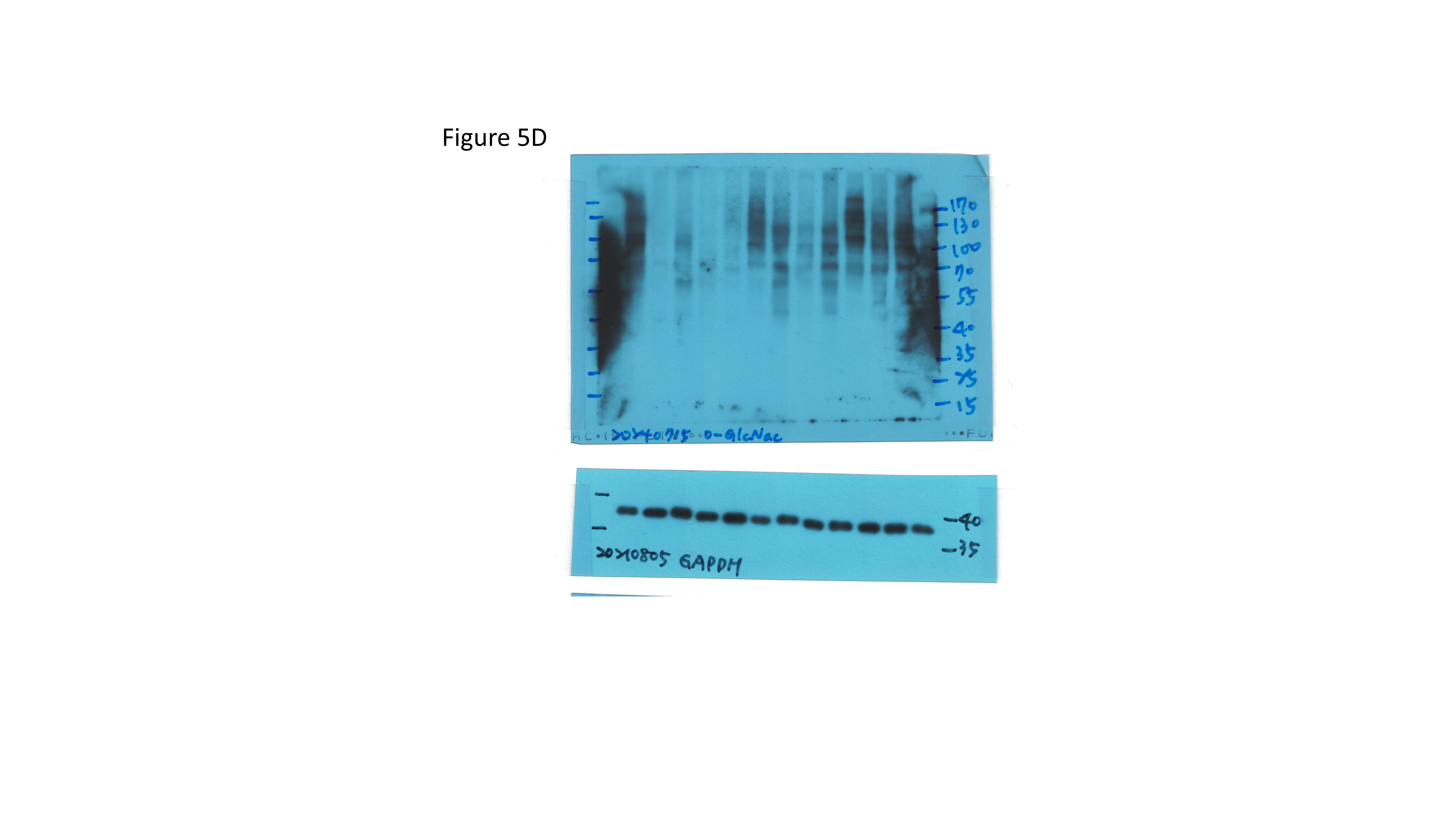

Supplement: Supplementary file 6 — Supplementary Material 6. [file 40348_2026_218_MOESM6_ESM.png]

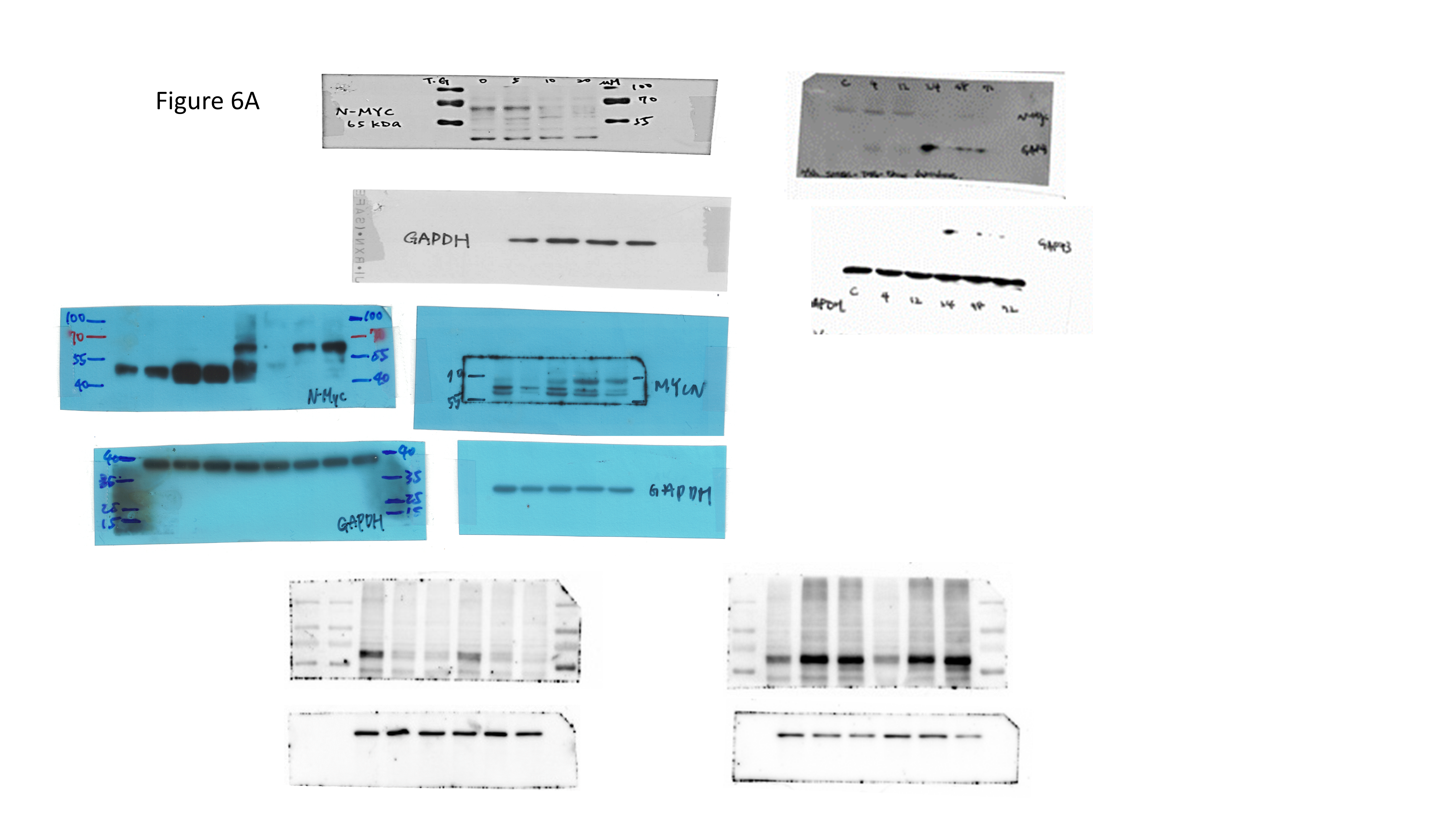

Supplement: Supplementary file 7 — Supplementary Material 7. [file 40348_2026_218_MOESM7_ESM.png]

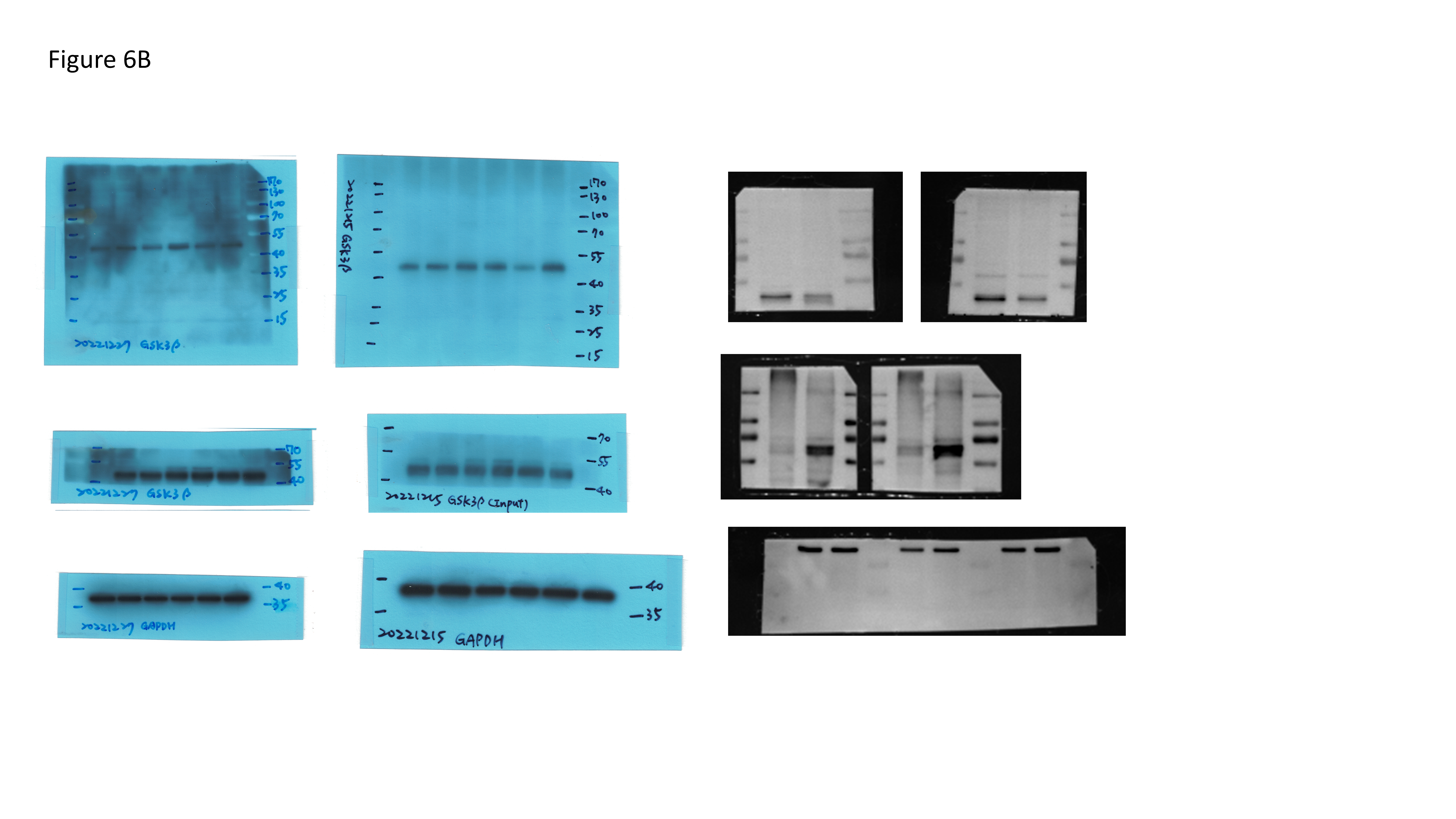

Supplement: Supplementary file 8 — Supplementary Material 8. [file 40348_2026_218_MOESM8_ESM.png]
